# Activity of the Heat Shock Protein 90 Inhibitor Ganetespib in Melanoma

**DOI:** 10.1371/journal.pone.0056134

**Published:** 2013-02-13

**Authors:** Xinqi Wu, Melina E. Marmarelis, F. Stephen Hodi

**Affiliations:** 1 Department of Medical Oncology, Dana-Farber Cancer Institute and Harvard Medical School, Boston, Massachusetts, United States of America; 2 Melanoma Disease Center, Dana-Farber/Brigham and Women’s Cancer Center, Boston, Massachusetts, United States of America; 3 Harvard Medical School, Boston, Massachusetts, United States of America; The Moffitt Cancer Center & Research Institute, United States of America

## Abstract

Heat shock protein 90 (HSP90) is involved in the regulation of diverse biological processes such as cell signaling, proliferation and survival, and has been recently recognized as a potential target for cancer therapy. Ganetespib is a potent ATP competitive inhibitor of HSP90. Ganetespib downregulated the expression of multiple signal transducing molecules including EGFR, IGF-1R, c-Met, Akt, B-RAF and C-RAF, resulting in pronounced decrease in phosphorylation of Akt and Erk1/2 in a panel of five cutaneous melanoma cell lines including those harboring B-RAF and N-RAS mutations. Ganetespib exhibited potent antiproliferative activity on all five of these cell lines, with IC50 values between 37.5 and 84 nM. Importantly, Ganetespib is active on B-RAF mutated melanoma cells that have acquired resistance to B-RAF inhibition. Ganetespib induced apoptosis and cell cycle arrest at G1 and/or G2/M phase. Ganetespib induced cell cycle arrest was accompanied by altered expression of cyclin-dependent kinase inhibitor (CDKI) p21^Cip1^ and p27^Kip1^, cyclins B1, D1 and E, and/or cyclin-dependent kinases 1, 2 and 4. HSP90 is functionally important for melanoma cells and HSP90 inhibitors such as ganetespib could potentially be effective therapeutics for melanoma with various genetic mutations and acquired resistance to B-RAF inhibition.

## Introduction

Heat shock protein 90 (HSP90) is a ubiquitous molecular chaperone that promotes the conformational maturation and stabilization of numerous client proteins. HSP90 is constitutively expressed and can be upregulated during cellular stress [1]. Inhibition of HSP90 results in increased degradation of client proteins via the ubiquitin proteasome pathway [2]. HSP90 is involved in the regulation of diverse biological processes including cell signaling, proliferation, and survival, as many HSP90 clients are conformationally labile signaling molecules and recognized as oncoproteins [2]-[4]. Interactions with client proteins enable HSP90 to promote cancer cell growth and survival by supporting proliferative and/or anti-apoptotic mechanisms [2], [5], [6]. HSP90 has recently been recognized as a potential therapeutic target for cancer, as accumulation of over-expressed and mutated client proteins has been shown to promote a shift to the active and super-chaperone complex form of HSP90 in cancer cells, conferring a greater sensitivity of malignant cells to the loss of HSP90 function [7]. HSP90 as target for cancer therapy has potential advantages. It may represent a relatively stable target for drug treatment as no resistance mutations have been identified in this molecule thus far [8]. HSP90 inhibition has the potential to affect multiple signaling pathways that frequently contribute to the tumor development and progression [2].

Ganetespib is a novel and potent HSP90 inhibitor binding to the adenosine triphosphate (ATP)-binding domain of HSP90 [9]. It has been shown to induce degradation of multiple HSP90 client proteins, kill a wide variety of human cancer cell lines at low nanomolar concentrations *in vitro*, and exhibit potent anticancer activity in xenograft tumor models in mice [9]-[12].

Melanoma is the fifth and sixth most common cancer in men and women, respectively, in the United States [13]. Metastatic melanoma is one of the most aggressive forms of skin cancer with low response rate to standard chemotherapy and a median overall survival less than one year [14]. While the response rate of patients with BRAF V600E mutant metastatic melanoma to oral BRAF inhibitor vemurafenib is high, the median overall survival is approximately sixteen months [15]. The majority of the patients who initially responded acquired resistance to vemurafenib within months of initial treatment. Novel therapies are needed for effective treatment of melanoma. Ganetespib has potent antiproliferative activity on a panel of cutaneous melanoma cell lines through altering the expression of multiple regulators of growth and survival signaling pathways, cell cycle and apoptosis. These alterations ultimately result in cell cycle arrest and apoptosis in melanoma cells.

## Results

### Ganetespib Downregulates Multiple Signaling Pathways in Melanoma Cells

**Figure 1:**
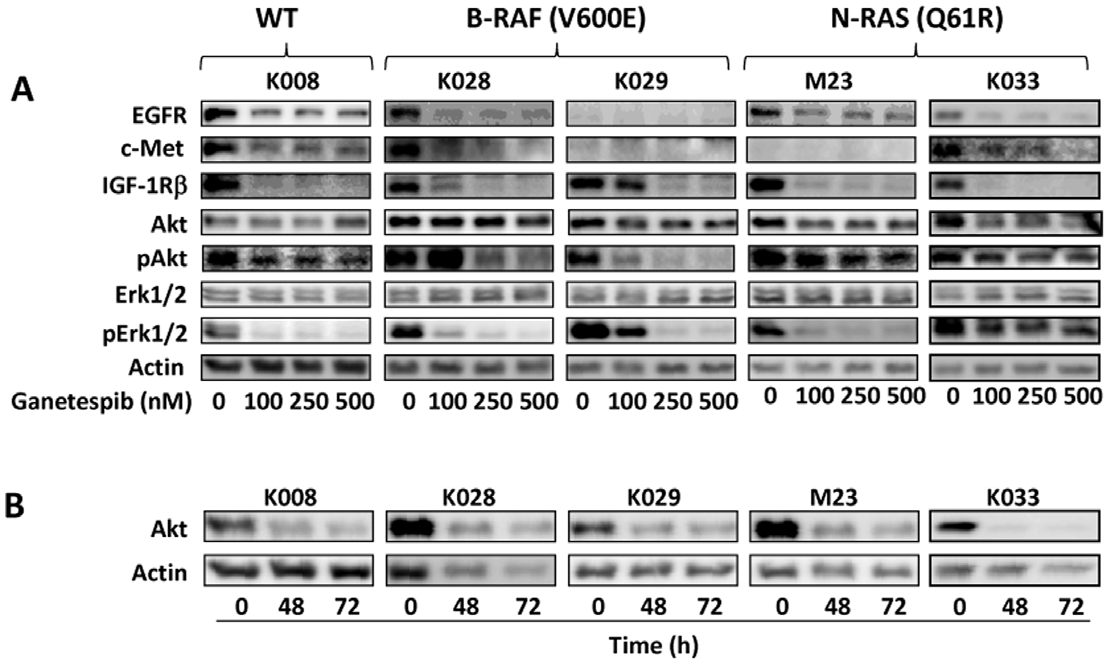
Downregulation of multiple signaling pathways by ganetespib in melanoma cells. A. Cells were treated with indicated amounts of ganetespib for 24 h. B-RAF and N-RAS mutational status of each cell line is indicated. B. Cells were treated with 250 nM ganetespib for 48 and 72 h. Proteins levels were determined by Western blot analysis.

We investigated the effect of ganetespib on signaling molecules that have been reported to be the clients of HSP90, including c-Met, EGFR, and IGF-1R [2], [16], [17], in a panel of five melanoma cell lines (Figure 1A). Cell lines K028 and K029 harbor B-RAF V600E mutation while K033 and M23 carry N-RAS Q61R mutation. Cell line K008 is wild type for B-RAF and N-RAS. In agreement with our previous observations [18], melanoma cell lines express varying levels of EGFR, c-Met, and IGF-1Rβ. EGFR was expressed in K008, K028, M23 and K033 cells and downregulated after ganetespib treatment. c-Met expression was detected in K008, K028 and K033 cells and was decreased by ganetespib. IGF-1R was expressed in all melanoma cell lines and reduced by ganetespib. The effect of ganetespib on EGFR in K029 cells and c-Met in K029 and M23 cells remained unclear due to very low abundance of these receptors in these cells. We examined the effect of ganetespib on the expression of phosphorylated EGFR, c-Met and IGF-1R in K008 and K028 cells that express all three receptors. In line with decreased expression of these receptors, the levels of phosphorylated EGFR, c-Met and IGF-1R were reduced in ganetespib treated cells (data not shown). Akt was minimally decreased after 24 hours of ganetespib treatment (Figure 1A), but was substantially reduced after 48 h treatment (Figure 1B), in agreement with being a client protein of HSP90 [19]. Akt phosphorylation was decreased in all cell lines after ganetespib treatment for 24 h except it was increased in K028 cells treated with 100 nM ganetespib (Figure 1A). Erk1/2 phosphorylation was markedly inhibited in all cell lines while total Erk1/2 levels remained unaltered (Figure 1A).

### Ganetespib Decreases Viability of Melanoma Cells

**Figure 2:**
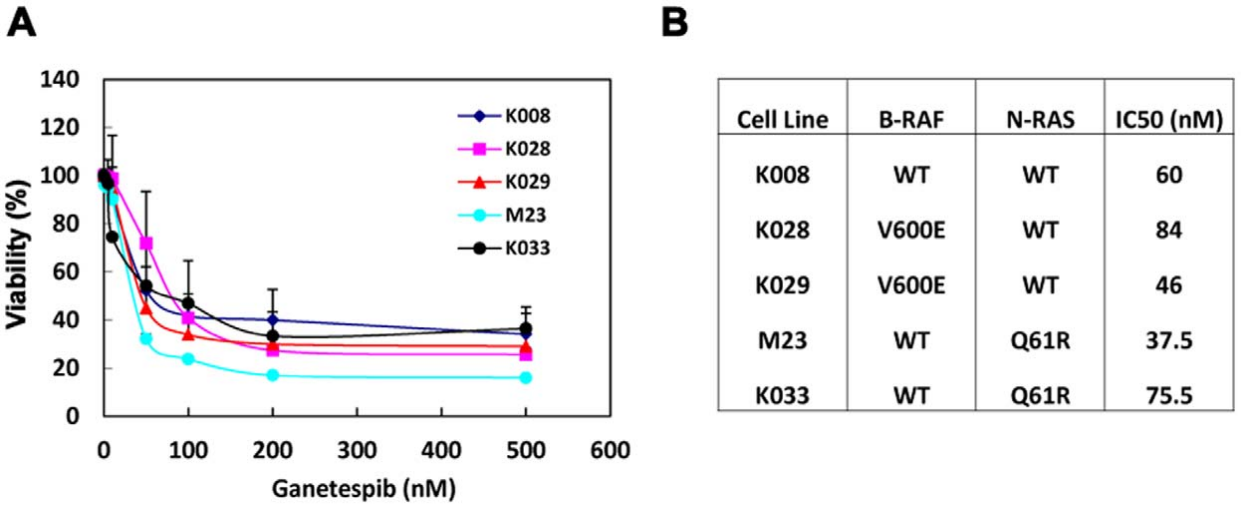
Antiproliferative action of ganetespib on melanoma cells. A. Ganetespib reduced viability. Cells were treated with varying amounts of ganetespib for 72 h and subjected to MTS assay. Data are expressed as mean±SD of three independent experiments. B. Mutational status and ganetespib IC50 of cell lines.

We next tested the cytotoxic effect of ganetespib on K008, K028, K029, M23 and K033 melanoma cells. Ganetespib effectively decreased viability of all five cell lines (Figure 2A). Maximum viability reduction, ranging from 60 to 90%, was achieved with 100 nM ganetespib. The IC_50_ values of ganetespib on these cell lines are 37.5 - 84 nM (Figure 2B).

### Ganetespib Induces Cell Cycle Arrest in Melanoma Cell Lines

**Figure 3:**
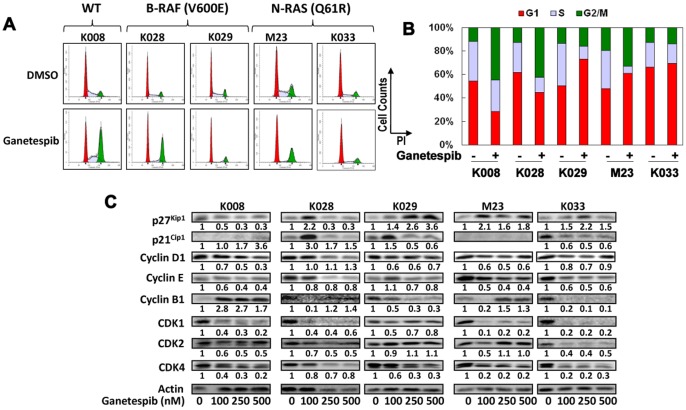
Ganetespib induced cell cycle arrest in melanoma cells. A. Cells were treated with 250 nM ganetespib for 24 hours, stained with PI and subjected to FACS analysis. B. Bar graphs of percentage of G1, S, and G2/M populations in control and ganetespib treated cells. C. Alterations in expression of multiple cell cycle regulating proteins induced by ganetespib. Cells were treated with indicated amounts of ganetespib for 48 h and analyzed by Western blot analysis. Relative expression levels of proteins (treated vs. control cells) are indicated.

In order to better understand the antiproliferative action of ganetespib on melanoma cells, we examined its effect on cell cycle progression. Treatment with 250 nM ganetespib for 24 hours resulted in cell cycle arrest at varying phases (Figure 3A). Substantial G2 arrest was observed in K008 and K028 cells, G1 arrest in K029 cells, and G1 and G2/M arrest in M23 cells (Figure 3B). A modest increase in G1 population was also seen in ganetespib treated K033 cells.

### Effect of Ganetespib on Cell Cycle Regulators

To better understand the molecular mechanisms by which ganetespib induced cell cycle arrest, we examined the expression of negative and positive cell cycle regulators in melanoma cells treated with ganetespib for 48 hours (Figure 3C). The expression of cyclin-dependent kinase inhibitor (CDKI) p27^Kip1^ was increased in K029, M23 and K033 cells. An increase in p27^Kip1^ was also detected in K028 cells treated with 100 nM ganetespib. The expression of p21^Cip1^ was elevated in K008 and K028 cells and in K029 cells treated with 100 nM ganetespib while it was reduced in K033 cells. The expression of p21^Cip1^ in M23 cells was below detectable levels. Cyclin D1 expression was reduced in K008, K029 and M23 cells and to a lesser extent in K033 cells treated with 100 and 250 nM ganetespib. Cyclin E was decreased in K008, M23 and K033 cells. A modest decrease in cyclin E was also seen in K028 and in K029 cells treated with 250 and 500 nM ganetespib. Cyclin B1 was increased in K008 cells and in M23 cells treated with 250 and 500 nM ganetespib while it was reduced in K029 and K033 cells and in K028 and M23 cells treated with 100 nM ganetespib. The expression of CDK1 was significantly reduced in K008, K028, M23 and K033 cells and to a lesser extent in K029 cells. The expression of CDK2 was reduced in K008, K028 and K033 cells and in M23 cells treated with 100 nm ganetespib. CDK4 was significantly reduced in K008, K029, M23 and K033 cells and modestly decreased in K028 cells.

### Ganetespib Induces Apoptosis in Melanoma Cells

**Figure 4:**
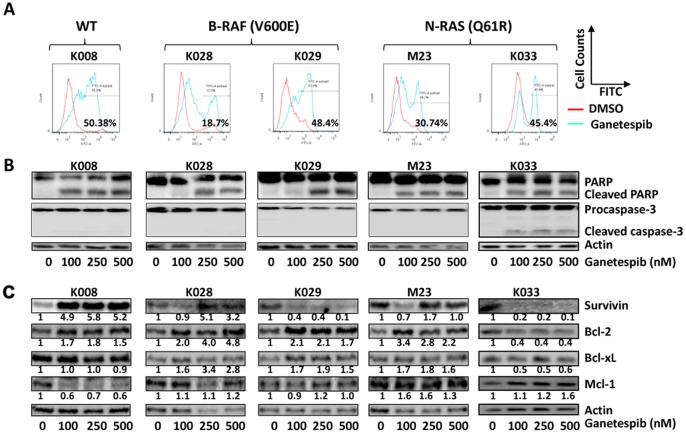
Ganetespib induced apoptosis in melanoma cells. Cells were treated with 100 nM ganetespib for 72 hours. A. Apoptotic cells were detected using Annexin V-FITC staining and FACS analysis. B. Cleavage of PARP and casapase-3. C. Effect of ganetespib on the expression of antiapoptotic proteins. Cells were treated with ganetespib for 48 h and analyzed using Western blot analysis. Relative expression levels of proteins are indicated.

Annexin V staining analysis revealed that ganetespib treatment significantly induced apoptosis in K008, K028, K029, M23 and K033 cells (Figure 4A). In agreement, ganetespib treatment resulted in PARP cleavage in all cell lines and caspase-3 cleavage in K033 cells (Figure 4B). Modest decrease in pro-caspase-3 levels was also seen in K028 and K029 cells (Figures 4B), although cleaved casapase-3 was not detected in these cell lines.

### Effect of Ganetespib on Anti-apoptotic Proteins

As ganetespib significantly induced apoptosis in melanoma cells, we next investigated the response of antiapoptotic proteins to ganetespib. Survivin was reduced in K029 and K033 cells, but induced in K008 and K028 cells (Figure 4C). Bcl-2 was decreased in K033 cells while it was increased in the rest of cell lines. Bcl-xL levels were elevated in K028, K029 and M23 cells but decreased in K033 cells while remained unaltered in K008 cells. Mcl-1 was decreased in K008 cells, increased in M23 and K033 cells, and unaltered in K028 and K029 cells.

### Effect of Ganetespib on B-RAF, C-RAF and N-RAS

**Figure 5:**
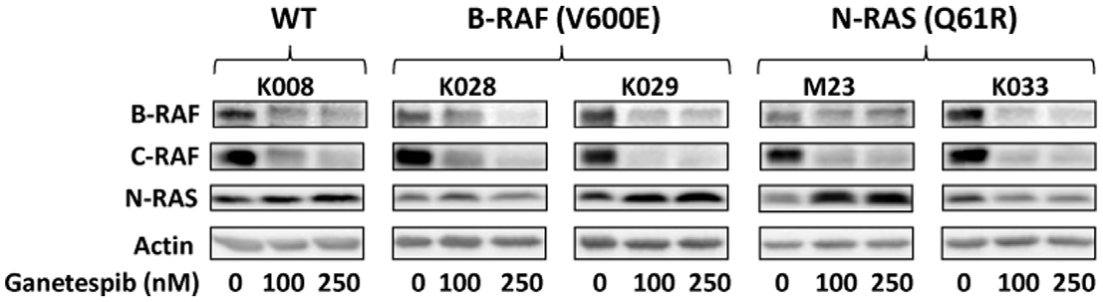
Effect of ganetespib on B-RAF, C-RAF and N-RAS expression in melanoma cells. Cells were treated with indicated amounts of ganetespib for 48 h and subjected to Western blot analysis.

Oncogenic B-RAF and N-RAS mutations have been detected in approximately 60% and 20% of cutaneous melanoma respectively [20]-[24]. While B-RAF inhibition is initially effective for melanoma carrying a B-RAF mutation, resistance to B-RAF inhibition develops within months after start of treatment [25], [26]. MAPK/Erk1/2 activation via C-RAF overexpression and upregulation of RTKs (PDGFRβ and IGF-1R) or N-RAS mutation have been reported to be among the mechanisms by which melanoma cells acquire resistance to B-RAF inhibitors [27]-[29]. Therefore, we examined whether ganetespib affected the expression of B-RAF, C-RAF and N-RAS in melanoma cells. Treatment with 100 and 250 nM ganetespib resulted in marked downregulation of wild type and mutant B-RAF and C-RAF expression in all 5 melanoma cells lines tested (Figure 5). In comparison, ganetespib increased N-RAS expression in three N-RAS wild type cell lines (K008, K028 and K029) and one N-RAS mutated cell line (M23) and modestly decreased N-RAS expression in one N-RAS mutated cell line (K033) (Figure 5).

### Ganetespib Effectively Reduces Viability of Melanoma Cells with Acquired Resistance to B-RAF Inhibition

**Figure 6:**
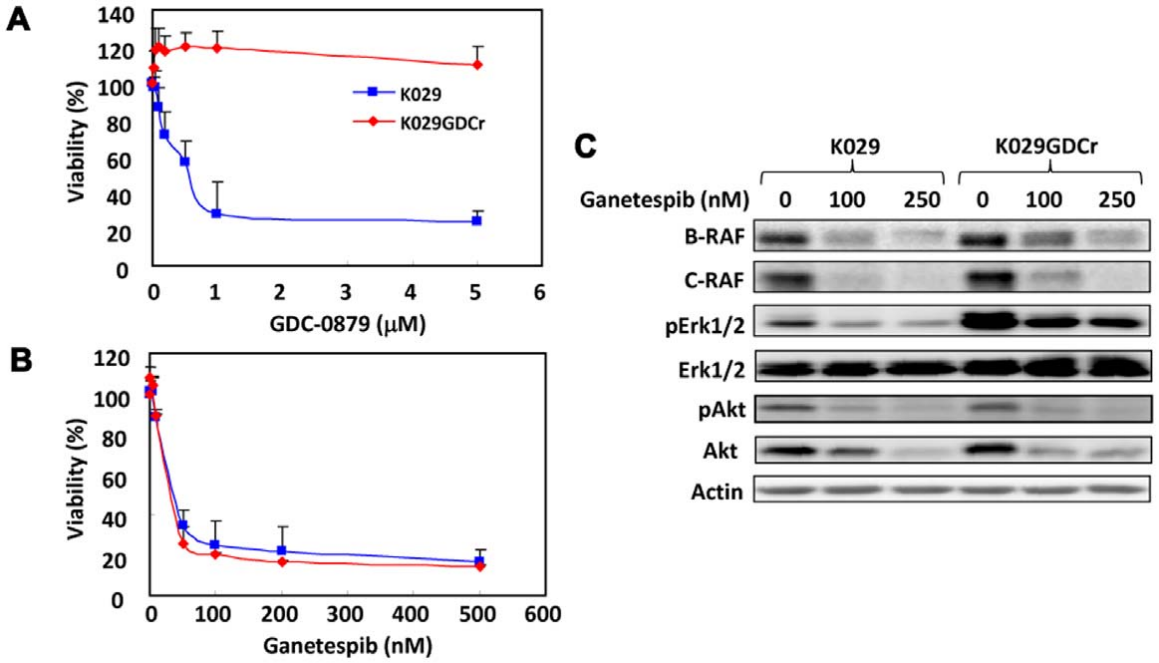
Ganetespib reduced viability of K029 cells with acquired resistance to B-RAF inhibition as effectively as the parental cells. A. Chronic treatment of K029 cells with B-RAF inhibitor GDC-0879 resulted in resistance to B-RAF inhibition. K029 cells and GDC-0879 resistant K029 cells (K029GDCr) were treated with varying amounts of GDC-0879 for 3 days and subjected to MTS assay. B. Ganetespib reduced viability of K029GDCr cells as effectively as K029 cells. K029GDCr (in the presence of 1 µM GDC-0879) and K029 cells were treated with ganetespib for 3 days and subjected to MTS assay. C. Ganetespib downregulated the expression of B-RAF, C-RAF, phosphorylated Akt and Erk1/2 in K029 and K029GDCr cells. Cells were treated with indicated amounts of ganetespib for 48 h and proteins were determined by Western blot analysis.

Currently, there is no effective treatment for melanoma with acquired resistance to B-RAF inhibition. We investigated whether ganetespib exhibited antiproliferative action on melanoma cells with acquired resistance to B-RAF inhibition. GDC-0879 is a B-RAF mutant specific inhibitor and has been shown to inhibit the growth of melanoma cells harboring B-RAF mutations [30]. As expected, GDC-0879 exerted pronounced antiproliferative activity to K029 cells at nanomolar concentrations (Figure 6A). After subjection to chronic treatment with GDC-0879, K029 cells developed resistance to GDC-0879 (Figure 6A). However, the viability of K029 cells with acquired resistance to GDC-0879 (K029GDCr cells) were reduced by ganetespib to similar levels as the parental K029 cells (Figure 6B). Resistance to GDC-0879 was associated with increased expression of B-RAF, C-RAF, and Erk1/2 activity (Figure 6C) in agreement with previous findings [27]-[29]. Ganetespib decreased the expression of B-RAF, C-RAF, and phosphorylation of Akt and Erk1/2 (Figure 6C).

## Discussion

The HSP90 inhibitor ganetespib, with IC50 values <100 nM, exhibited profound antiproliferative activity against a panel of cutaneous melanoma cells including those that carry B-RAF and N-RAS mutations, as well as those with acquired resistance to B-RAF inhibition. Ganetespib exerted its antiproliferative activity through induction of cell cycle arrest and apoptosis in association with inhibition of multiple tyrosine receptor kinases, B-RAF, C-RAF, and the AKT and Erk1/2 pathways.

The PI3K/Akt and MAPK/Erk pathways are critical for melanoma cell growth and survival [31], [32] and were significantly inhibited by ganetespib. During the course of this study, similar effects of the HSP90 inhibitor XL888 on phosphorylation of Akt and Erk1/2 in melanoma cells have been reported [33]. Inhibition of these pathways may contribute to ganetespib induced growth inhibition and apoptosis as inhibition of these pathways, alone or in combination, reduced viability of K008 and K028 cells (data not shown) and induced apoptosis in melanoma cells [33]. In agreement with previous findings that Akt but not Erk1/2 was a client protein of HSP90 [19], [34], the expression of Akt but not Erk1/2 was reduced by ganetespib. Ganetespib may inhibit Akt phosphorylation through downregulating Akt expression and repress Erk1/2 phosphorylation through downregulating their upstream activating kinases (MEK and MEKK1), as MEK and MEKK1 are client proteins of HSP90 [35], [36]. Akt and Erk1/2 can be activated by signals from multiple tyrosine kinase receptors including EGFR, IGF-1R and c-Met [37]. In agreement with being client proteins of HSP90 [2], [16], [17], [38], the expression of these receptors was decreased by ganetespib. Downregulation of these receptors will also result in inhibition of Akt and Erk1/2 phosphorylation. Thus, ganetespib may inhibit Akt and Erk1/2 activation by targeting multiple cellular signaling processes. Small molecule inhibitors of c-Met, EGFR or IGF-1R reduced viability of K008 and K028 cells that express all these receptors (unpublished data), suggesting that these receptor tyrosine kinases may play a role in survival and growth of these cell lines and their inhibition may be relevant to the anti-melanoma activity of ganetespib.

Ganetespib induced cell cycle arrest at G1and/or G2/M phase in cell line dependent manner. Similar cell cycle effects were also observed with XL888 [33]. Ganetespib induced cell cycle arrest was associated with upregulation of negative cell cycle regulators (p21^Cip1^ and/or p27^Kip1^) and/or downregulation of positive regulators (cyclins D1 and E, CDK1, CDK2 and CDK4). This is in general in line with the roles of these regulators in cell cycle regulation [39], [40]. CDK1, CDK2 and CDK4 have been reported to be chaperoned by HSP90 [17], [41]-[43]. However, cyclin D1 and cyclin E are not considered to be a client protein for HSP90. The observed downregulation of cyclin D1 may result from downregulation of Akt and Erk1/2 pathways, which control cyclin D1 expression [44], [45]. Downregulation of cyclin E could result from decreased D-type cyclins, as the transcriptional activation of the cyclin E gene depends on the activity of D-type cyclins [46]. The upregulation of the CDK inhibitors p27^Kip1^ and p21^Cip1^ could be attributed to inhibition of the Akt and Erk pathways [40], [47]-[49]. Despite being reported to be a client protein of HSP90 [50] and downregulated in K029 and K033 cells, cyclin B1 was induced by ganetespib in two cell lines (K008 and M23) that were arrested at G2/M. Cyclin B1 activity is essential for progression from G2 into M phase. Binding of cyclin B1 to CDK1 allows CDK1 to be activated [51], [52]. The active cyclin B1-CDK1 complex translocates to the nucleus and phosphorylates nuclear substrates. These phosphorylation events are necessary for mitotic onset. The accumulation of cyclin B1 may allow these cells to progress through G1 and S phase and enter G2 phase. On the other hand, CDK1 was downregulated by ganetespib in all three cell lines (K008, K028 and M23) that were arrested at G2/M phase. In addition, p21^CIP1^ was upregulated in K008 and K028 cells and p27^KIP1^ was upregulated in M23 cells. Both p21^Cip1^ and p27^Kip1^ can block G2/M transition by direct interaction with B1 and block cyclin B1-associated kinase activities [52], [53]. These findings suggest that ganetespib may induce G2/M arrest by downregulating CDK1 in combination with accumulation of p21^CIP1^ or p27^Kip1^.

Ganetespib substantially induced apoptosis in all melanoma cell lines tested here. Although the common anti-apoptotic proteins survivin, Bcl-2, and Bcl-xL have been reported to be client proteins of HSP90 [18], [54], [55], they were, surprisingly, not altered or even induced by ganetespib in most of the cell lines. Similar response to ganetespib was also observed with antiapoptotic protein Mcl-1. Ganetespib only decreased the expression of survivin in K029 and K033 cells, Bcl-2 and Bcl-xL in K033 cells, and Mcl-1 in K008 cells. It is widely believed that inhibition of Hsp90 with small molecule inhibitors can disrupt the physical binding of survivin to Hsp90, leading to survivin downregulation [54]. However, we showed here that survivin protein levels were increased in K008 and K028 cells treated with ganetespib. Although the molecular events for this increase is not clear, it has been reported that survivin expression was induced by HSP90 inhibitors in some cancer cell lines via cell context dependent transcriptional, translational and/or post-translational (such as 26S proteasome-mediated protein degradation) mechanisms [56]. These findings suggest that ganetespib-induced apoptosis is largely attributed to altered expression of other pro- and/or anti-apoptotic proteins that remain to be identified. Ganetespib induced upregulation of p27^Kip1^ may play a role in apoptosis induction as p27^Kip1^ has been shown to induce apoptosis [57]-[59]. The proapoptotic BIM proteins have also been recently shown to be induced by XL888 and play a role in XL888 induced apoptosis in melanoma cells [33].

Ganetespib profoundly inhibited the growth of melanoma cells harboring wild type and mutated B-RAF or N-RAS. B-RAF and N-RAS mutations play a key role in the development of human melanomas [20]-[22]. B-RAF mutations have been found in approximately 50% of human melanomas with V600E being the most common mutation [20]. B-RAF V600E stimulates constitutive activation of MEK/Erk/12 pathway, resulting in growth factor independent proliferation [21]-[23]. In agreement with being clients of HSP90 [60]-[62], the expression of both wild-type and mutant B-RAF was decreased by ganetespib in all melanoma cell lines including those cells with acquired resistance to B-RAF inhibition. Similar to B-RAF, the expression of C-RAF was reduced in ganetespib treated cells. Downregulation of B-RAF and C-RAF contributes to inhibition of Erk1/2 phosphorylation and the growth of melanoma cells including those with acquired resistance to B-RAF inhibition. Interestingly, although ganetespib exerted antiproliferative activity towards melanoma cells harboring mutated N-RAS, N-RAS was induced by ganetespib in most of the cell lines tested. To the best of our knowledge, N-RAS has not been shown to be a client of HSP90.

In melanoma cells carrying the B-RAF mutations, activation through B-RAF and subsequent downstream signaling is the major driving force for tumor progression, making B-RAF an attractive target for anti-melanoma therapy. Clinical data has shown that treatment with B-RAF inhibitor vemurafenib resulted in tumor shrinkage and median progression-free survival for greater than six months in patients with B-RAF V600E mutated melanoma [25]. However, the majority of the patients who initially responded developed resistance to vemurafenib. MAPK/Erk1/2 activation via C-RAF overexpression and upregulation of RTKs (PDGFRβ and IGF-1R) or N-RAS mutation are among the mechanisms for acquired resistance to B-RAF inhibition [27]-[29]. Ganetespib inhibited the growth of melanoma cells with acquired resistance to B-RAF inhibition as effectively as the parental cells. Similar findings have recently reported with HSP90 inhibition with XL888 [33]. These findings suggest that ganetespib may potentially be used for patients with melanoma resistant to B-RAF inhibition. Ganetespib may prevent melanoma cells from acquiring resistance to B-RAF inhibition by targeting multiple signal pathways and kinases important for development of resistance to B-RAF inhibitors.

The present study has its limitations. For example, the data presented were obtained using in vitro models of melanoma and in vivo studies to examine anti-melanoma activity of ganetespib are important. Furthermore, the molecular responses of melanoma cells to ganetespib and the mechanisms by which ganetespib induced cell cycle arrest and apoptosis have not been fully investigated. Nonetheless, our data show that ganetespib exerts potent antiproliferative activity against a panel of melanoma cell lines including those with common activating mutations. Inhibition of HSP90 function by ganetespib produced complex molecular effects in melanoma cells. Some of the molecular effects of HSP90 inhibition were similar among the melanoma cell lines tested. This is exemplified by downregulation of c-Met, IGF-1R, EGFR, Akt, phosphorylation of Akt and Erk1/2, suppression of positive cell cycle regulators and/or upregulation of negative cell cycle regulators. These shared molecular events were translated into a similar pattern of biologic consequences such as cell cycle arrest and apoptosis. However, the degree of these effects varied among the cell lines. Furthermore, distinct effects of ganetespib on the expression of some cell cycle and apoptosis regulatory proteins were observed among the cell lines. These findings reflect tumor heterogeneity and may influence the phase and degree of cell cycle arrest and death. These complex effects of HSP90 inhibition may provide optimal anti-tumor activity and prevent further development of resistance**.** These findings underscore the therapeutic potential of HSP90 inhibitors such as ganetespib for melanoma.

## Materials and Methods

### Cell Lines

Cutaneous melanoma cell lines K008, K028, K029, K033 and M23 were established from harvested fresh tissues that underwent mechanical and enzymatic digestion and *in vitro* expansion. Tumor samples were obtained from patients on Dana-Farber/Harvard Cancer Center Institutional Review Board approved protocols with written informed consent for the original human work that produced the tissue samples. Cutaneous melanoma cells were grown in DMEM containing 10% FBS, 50 µg/ml penicillin and 100 µg/ml streptomycin.

### Viability Assay

Cells were seeded in 96-well plates at 2x10^3^ cells per well and incubated over night followed by treatment with varying amount of Ganetespib (provided by Synta Pharmaceuticals Corp.) for 72 h. Cell viability was determined using MTS assay per manufacturer instructions (Promega, Madison, WI).

### Cell Cycle and Apoptosis Analysis

After treatment with Ganetespib, cells were harvested by trypsinization and analyzed for cell cycle distribution and apoptosis as previously described [18]. For cell cycle analysis, cells were fixed in ethanol, stained with propidium iodide in PBS containing Triton X-100 (0.1%) and RNase A (0.2 mg/ml) for 30 min, and then subjected to FACS analysis. For apoptosis analysis, cells were incubated with FITC-Annexin V in 1x Annexin Binding buffer (BD Bioscience, San Joes, CA) for 15 min and subjected to FACS analysis. Cell cycle distribution and percentage of apoptotic cells were estimated using ModFit and Flowjo software respectively.

### Immunoblot Analysis

Whole cell lysate preparation and immunoblot analysis were performed as described previously [18]. Antibodies against EGFR, c-Met, IGF-1R, Akt, phospho-Akt, Erk1/2, phospho-Erk1/2, cyclin D1, cyclin B1, Bcl-2, Bcl-xL, survivin, CDK2 and C-RAF were purchased from Cell Signaling Technology (Danvers, MA). Antibodies against P27^Kip1^, p21^Cip1^, CDK1, CDK4, cyclin E, B-RAF were purchased from Santa Cruz Biotechnology (Santa Cruz, CA). N-RAS antibody was purchased from Millipore (Billerica, MA). Actin antibody was purchased from Sigma-Aldrich (St. Louis, MI). Density of protein bands was measured using NIH ImageJ software and normalized to that of actin.

### Establishment of Melanoma Cells Resistant to B-RAF Inhibition

K029 cells were treated with gradually increasing amounts (0.1, 0.2, 0.5, 1, 5 and 10 µM) of B-RAF V600E specific inhibitor GDC-0879 through ∼3 months until they were resistant to 10 µM of the drug. GDC-0879 was purchased from Selleck Chemicals (Houston, TX).
