# Correction: Activity of the Heat Shock Protein 90 Inhibitor Ganetespib in Melanoma

**DOI:** 10.1371/journal.pone.0309769

**Published:** 2024-08-29

**Authors:** Xinqi Wu, Melina E. Marmarelis, F. Stephen Hodi

After this article [1] was published, concerns were raised about Figs 1 and 3. Specifically:

- The Akt K028 and Akt M23 panels in Fig 1B appear similar.
- The Cyclin D1 M23 and Cyclin D1 K033 panels in Fig 3C appear similar.

In response, the corresponding author stated that in Fig 1B, the AKT immuno blot for M23 was incorrectly duplicated from that for K028, and in Fig 3C, the image of K033 cyclin D1 was accidentally copied from that of M23 during production of the figures. The updated versions of Figs 1B and 3C are provided here with the correct panels for Akt M23 in Fig 1B and Cyclin D1 K033 in Fig 3C from the original experiments. The corresponding author stated that the values underneath the Cyclin D1 K033 blot image in Fig 3C correspond to the correct Cyclin D1 K033 blot panel. The underlying blots for the Akt K029, K028, M23, and K008 panels in Fig 1B and the Cyclin D1 panels in Fig 3C from the original experiments are provided here (S1–S2 Files).

A member of the *PLOS ONE* Editorial Board reviewed the updated figures and stated that the revised figures support the original figures in [1]. The *PLOS ONE* Editors are satisfied that the data provided (S1–S2 Files) appear to support the published results.

The data underlying the remainder of the results in [1] are available from the corresponding author.

**Fig 1:**
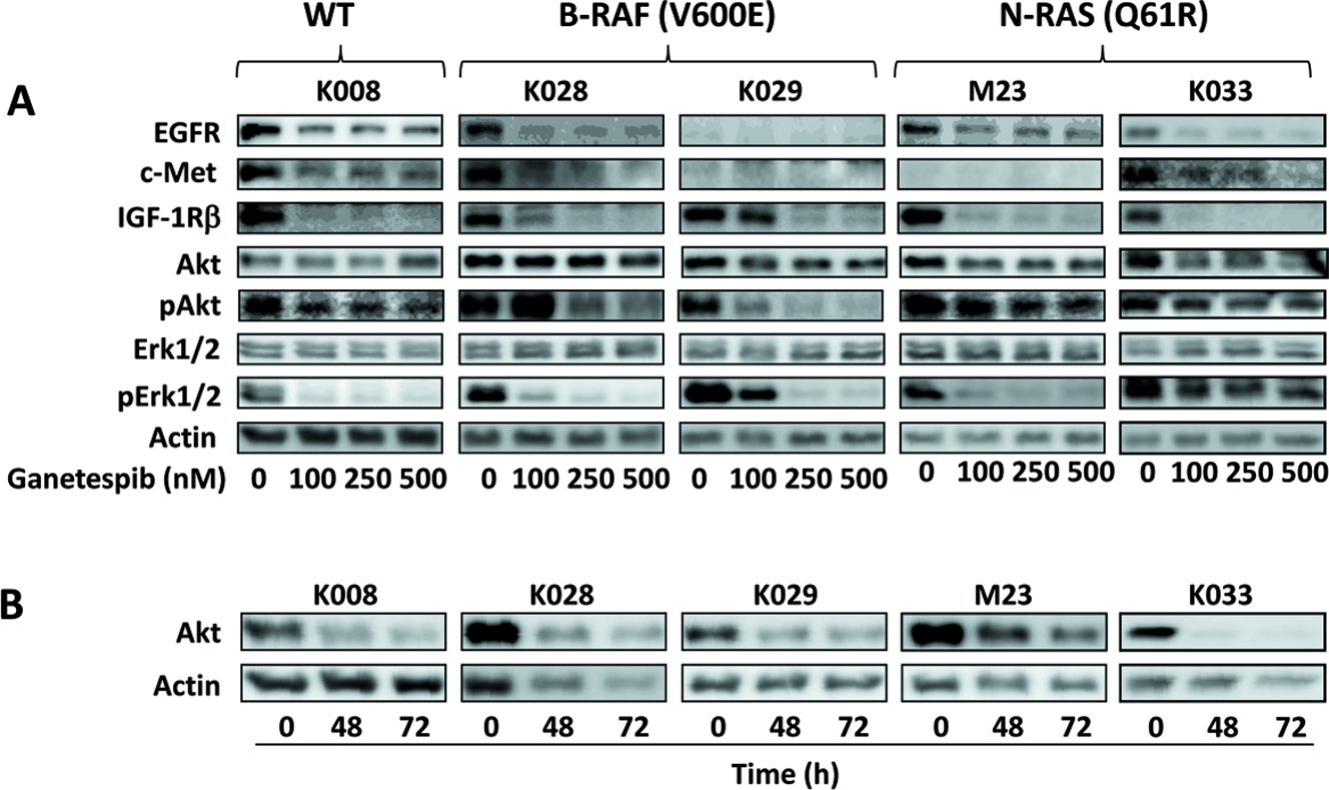
Downregulation of multiple signaling pathways by ganetespib in melanoma cells. A. Cells were treated with indicated amounts of ganetespib for 24 h. B-RAF and N-RAS mutational status of each cell line is indicated. B. Cells were treated with 250 nM ganetespib for 48 and 72 h. Proteins levels were determined by Western blot analysis.

**Fig 3:**
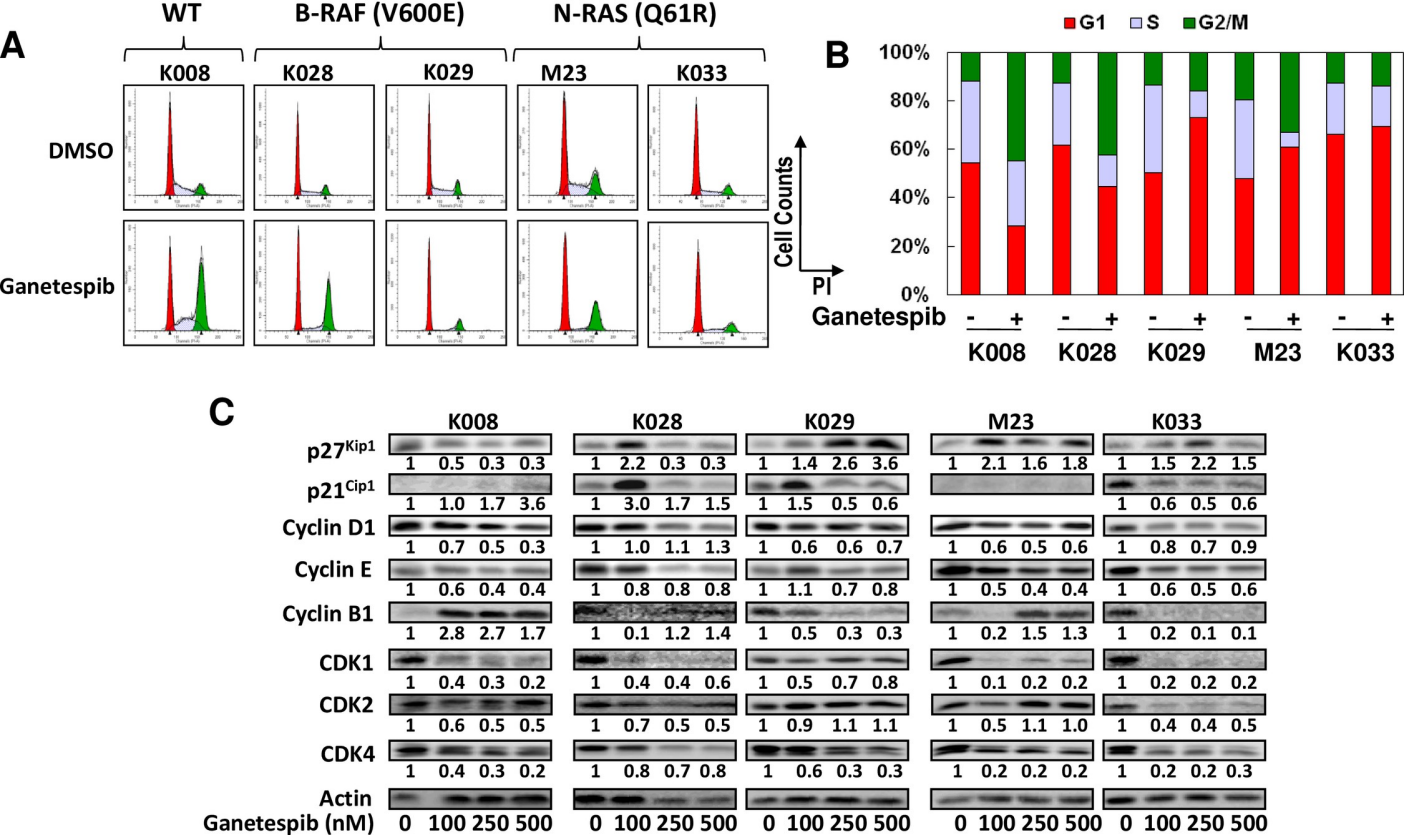
Ganetespib induced cell cycle arrest in melanoma cells. A. Cells were treated with 250 nM ganetespib for 24 hours, stained with PI and subjected to FACS analysis. B. Bar graphs of percentage of G1, S, and G2/M populations in control and ganetespib treated cells. C. Alterations in expression of multiple cell cycle regulating proteins induced by ganetespib. Cells were treated with indicated amounts of ganetespib for 48 h and analyzed by Western blot analysis. Relative expression levels of proteins (treated vs. control cells) are indicated.

## Supporting information

S1 File Underlying blots supporting the Akt K029, K028, M23 and K008 panels in Fig 1B in [1]. The membrane was probed with actin antibody first and then stripped and probed with Akt antibody. The lower sets of bands are the remaining signals of actin, however, the Actin bands shown in Fig 1B were taken from the image of blotting with actin antibody and not from the image blotting with AKT antibody. The K033 bands in Fig 1B are from a separate blotting.
(TIF)

S2 File Underlying blots supporting the Cyclin D1 panels in Fig 3C in [1]. (TIF)
