# Supplementary figures and images for: Correction: Activity of the Heat Shock Protein 90 Inhibitor Ganetespib in Melanoma
Source: PLoS One. 2024 Aug 29;19(8):e0309769. doi: 10.1371/journal.pone.0309769 (PMC11361599; doi:10.1371/journal.pone.0309769)

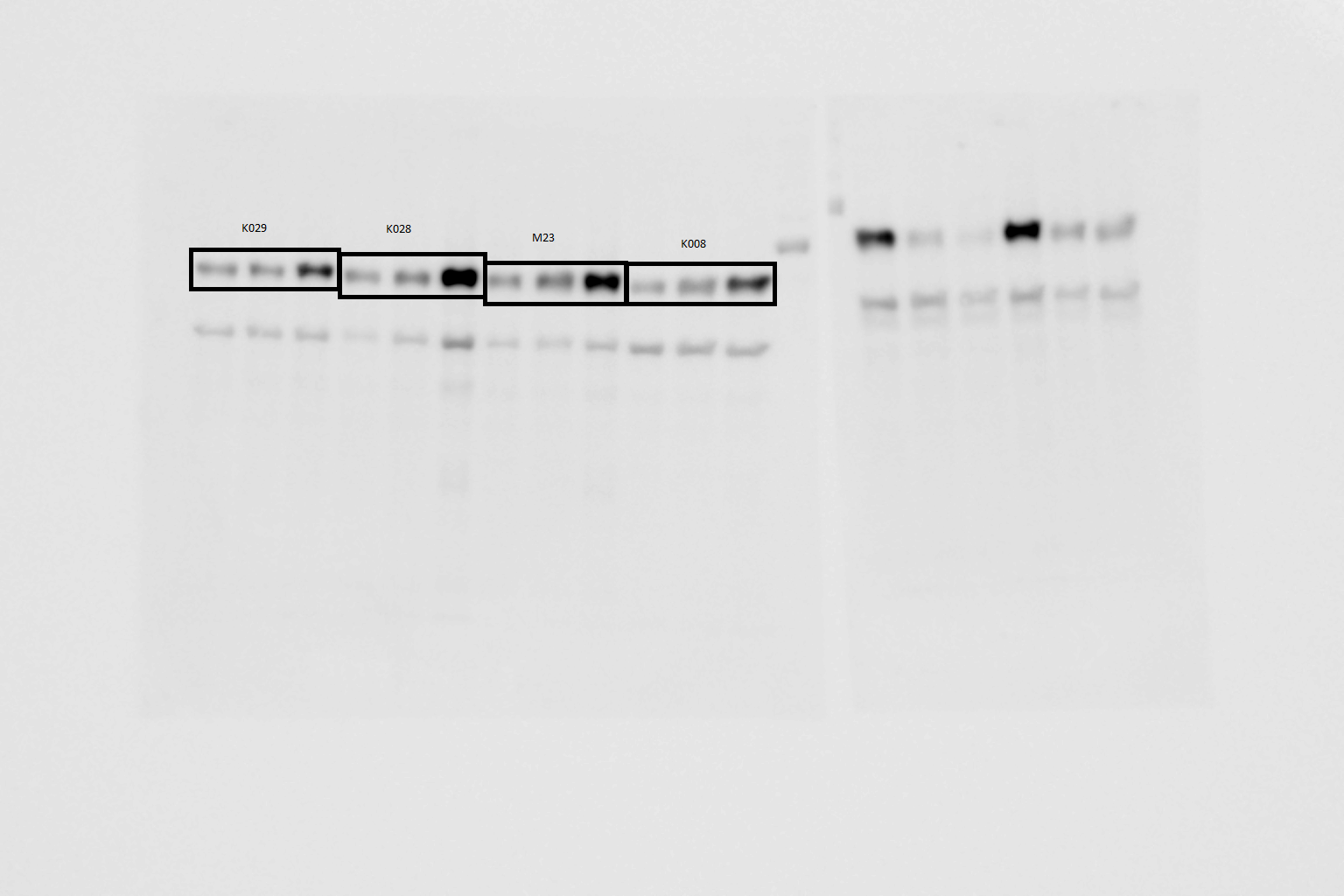

Supplement: S1 File — The membrane was probed with actin antibody first and then stripped and probed with Akt antibody. The lower sets of bands are the remaining signals of actin, however, the Actin bands shown in Fig 1B were taken from the image of blotting with actin antibody and not from the image blotting with AKT antibody. The K033 bands in Fig 1B are from a separate blotting. (TIF) [file pone.0309769.s001.tif]

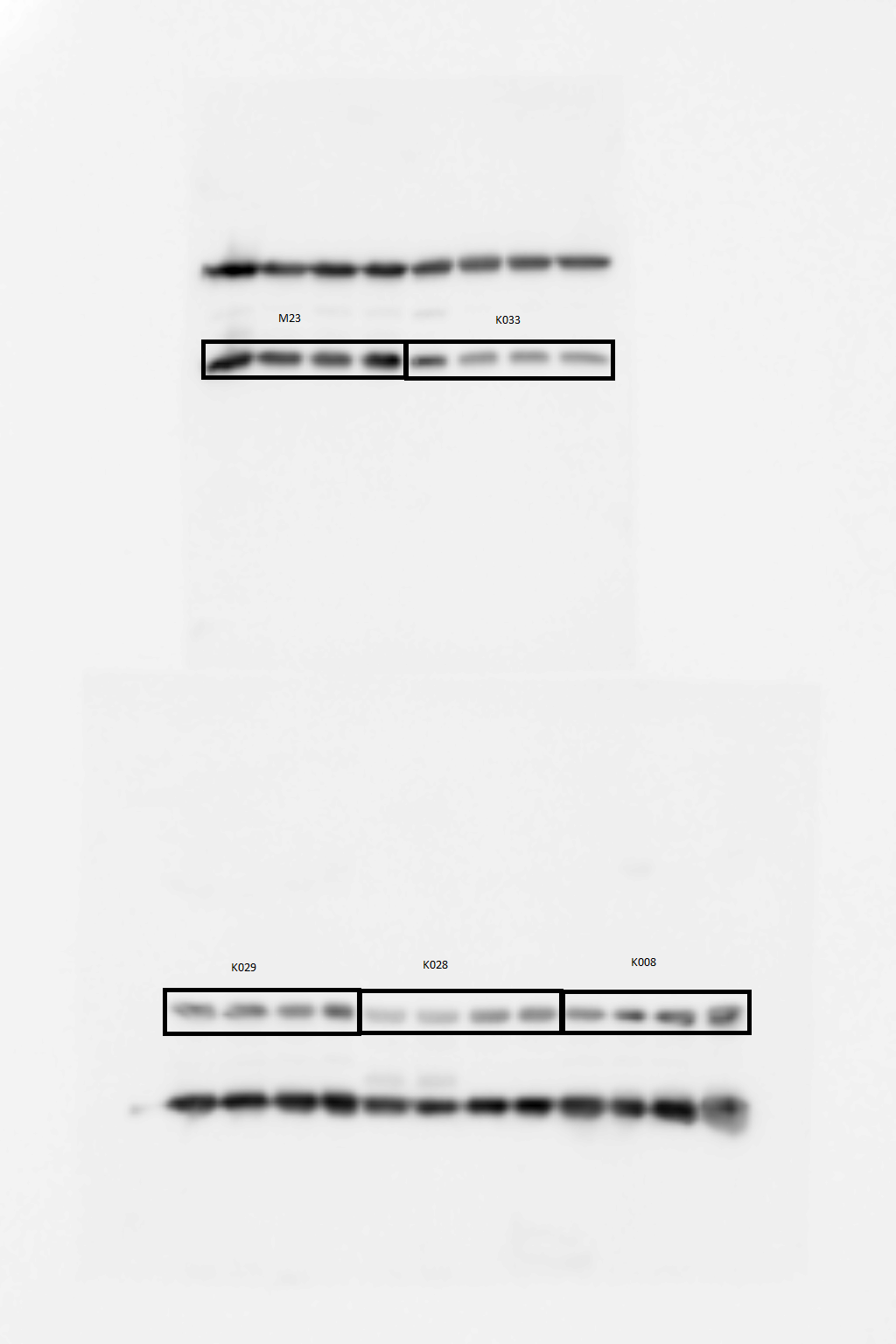

Supplement: S2 File — (TIF) [file pone.0309769.s002.tif]
